# WPSAR celebrates World Field Epidemiology Day

**DOI:** 10.5365/wpsar.2021.12.3.905

**Published:** 2021-09-07

**Authors:** Ashley Arashiro, Roxanne Andaya, Don Rivada, Michelle McPherson

**Affiliations:** a WPSAR Editorial Team, WHO Health Emergencies Programme, World Health Organization Regional Office for the Western Pacific, Manila, Philippines.

Today, 7 September 2021, is the inaugural World Field Epidemiology Day – a global movement to recognize and raise awareness of the vital role of field epidemiologists in protecting the health of populations and in advancing global health security. (*1*) World Field Epidemiology Day is coordinated by the Training Programs in Epidemiology and Public Health Interventions Network (TEPHINET), a professional network of 75 field epidemiology training programmes (FETPs) working across more than 100 countries. WPSAR provides a platform for sharing field epidemiology work in the Western Pacific Region; (*2*) it has partnered with TEPHINET to celebrate World Field Epidemiology Day.

In this editorial, we summarize our efforts to support and promote field epidemiology in the Western Pacific Region. WPSAR is also contributing to a series of TEPHINET webinars on Scientific Publishing for Field Epidemiologists, which cover converting a field report to a journal article, the journal submission process, conducting a peer review and responding to peer reviewer comments. We also acknowledge the special supplement of the International Journal of Infectious Diseases that honours World Field Epidemiology Day and documents the contributions of FETP within the public health architecture and in the response to public health events. (*3*)

Since 2010, WPSAR has strongly supported field epidemiology in the Region by publishing outbreak investigations, analyses of surveillance data and field epidemiology studies. These non-traditional article types align with FETP outputs. WPSAR also supports first-time and early career authors during pre-submission, peer review and publication and builds capacity in scientific writing by conducting workshops for FETPs in our Region. This support has clear benefits for the Region by facilitating timely information sharing for decision-making, in line with the Asia Pacific Strategy for Emerging Diseases and Public Health Emergencies. (*4*)

## Publications

As of 25 August 2021, WPSAR has published 367 articles, including reports on major public health events and emergencies such as Typhoon Haiyan, (*5*) the Great East Japan Earthquake (*6*) and outbreaks of infectious diseases in the Region such as influenza, Middle East respiratory syndrome (MERS), dengue, measles, tuberculosis and, most recently, coronavirus disease 2019 (COVID-19). (*7*) Many articles in WPSAR cover topics from field epidemiology investigations, with a range of article types (**Table 1**). Of the 103 Original Research articles, 18 described outbreak investigations and 40 used surveillance data for operational research; the remaining 45 described research projects, many of which used field epidemiology methods.

**Table 1:** Types of articles published in WPSAR

Article type	Total publications (*n* = 367)	Published by FETP affiliates (*n* = 62)	Published after WPSAR workshop participation (*n* = 44)
**Original Research**	**103**	**9**	**8**
Outbreak Investigation Report	46	29	20
Surveillance Report	42	6	3
Perspective	35	1	1
Brief Report	35	5	5
Lessons from the Field	22	2	4
Regional Analysis	19	-	-
Editorial	14	-	-
Field Investigation Report	13	4	-
Surveillance System Implementation/Evaluation	10	-	1
Letter to the Editor	8	3	1
Case Report/Case Series	7	1	-
Risk Assessment	7	2	1
Other	6	-	-

**Figure 1:**
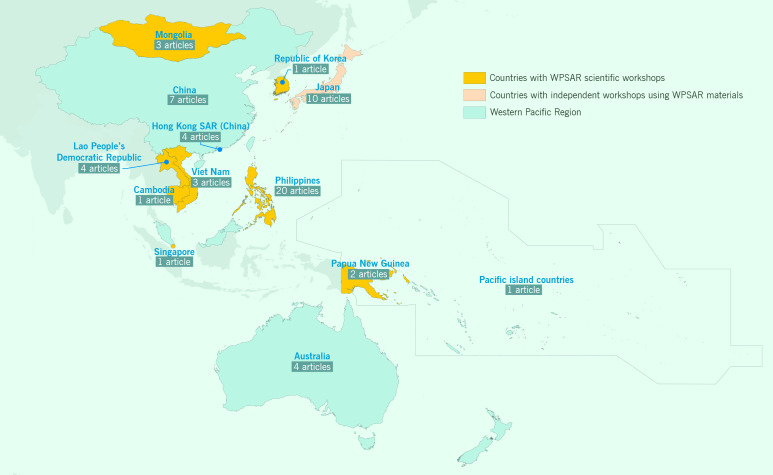
Map of the WHO Western Pacific Region showing FETPs that have published articles in WPSAR and the location of WPSAR scientific writing workshops.

Among the published WPSAR articles, 62 have authors whose affiliations at the time of publication were FETPs, either as fellows or staff members. (*8*) Authors of other WPSAR articles are also recognized as FETP fellows and graduates, highlighting the strong links between WPSAR and FETPs in our Region. There have been publications from 13 FETPs within the Western Pacific Region, plus one from FETP India that was submitted after being presented at a TEPHINET conference  (**Fig. 1**). These articles discussed outbreaks of infectious diseases such as measles, syphilis, mumps, chikungunya, MERS and foodborne gastroenteritis across a range of article types (**Table 1**). Examples of other topics were FETPs, surveillance systems and disaster responses.

[**insert** Figure 1]

## Scientific writing workshops

In the WPSAR scientific writing workshops, participants are introduced to the components of scientific writing and then draft a manuscript from their own field epidemiology project. These workshops are highly interactive, with exercises incorporating real-world examples of manuscripts that were submitted to WPSAR and revised for publication.

Participants include graduates, current fellows and training coordinators of FETPs, as well as ministry of health and WHO country office staff, ranging from 10 to 30 participants per workshop. Participants work on their own manuscript with the assistance of one or more facilitators. Since its inception in 2010 and up until the COVID-19 pandemic, WPSAR has conducted 20 of these workshops, attended by a total of more than 200 participants from eight countries (**Fig. 1**). Additionally, the Japan FETP ran several workshops using the WPSAR workshop materials.

WPSAR also facilitated scientific writing workshops at the biregional TEPHINET conferences in Viet Nam in 2013, Cambodia in 2016 and the Lao People's Democratic Republic in 2018. These workshops provided general scientific writing guidance and practical tips on converting field reports into journal articles to more than 100 conference participants, all of whom were FETP fellows or graduates.

The workshop evaluations indicated that participants liked that the workshops were practical and used real-life examples; they also liked being able to write up their own field epidemiology projects as scientific manuscripts. Participants found it very useful to have individual writing time and feedback sessions with the facilitator, and they were motivated to have their work published, albeit with the support of a co-author. Although there are many barriers to publishing articles, as evidenced by the low proportion of articles from the workshop that have been published in WPSAR (about 20%), participants reported that the workshop improved their skills in scientific writing and understanding the components of a scientific journal article. Barriers to publishing articles included having work schedules that do not allow for writing time, no perceived benefits to publishing articles, and difficulties writing in English and getting support from busy co-authors; similar barriers have been encountered elsewhere. (*9*)

A total of 44 articles have been published after being worked on at WPSAR workshops (**Table 1**). More than half of these published articles were from the workshop held in 2015 to support the special issue on the response to Typhoon Haiyan in the Philippines. (*10*) Many first-time authors published articles describing their roles in the public health response to this national disaster, which “enabled authors within the Philippines to learn how to write scientific papers and to provide a local perspective for their publications.” (*11*)

## Conclusion

The WPSAR Editorial Team, seven of whom are FETP graduates themselves, are pleased to be supporting the inaugural World Field Epidemiology Day. To ensure that our submissions are reviewed by field epidemiology practitioners, we encourage all FETP fellows, graduates and staff from the Western Pacific Region to register as reviewers for WPSAR. We look forward to continuing to publish field epidemiology projects and supporting the work of FETPs in the Western Pacific Region.

## References

[1] World Field Epidemiology Day [website]. Decatur, GA: Training Programs in Epidemiology and Public Health Interventions Network (TEPHINET); 2021. Available from: https://www.worldfieldepidemiologyday.org/

[2] Field E, Kasai T. Western Pacific Surveillance and Response: a journal to reflect the needs of our Region. West Pac Surveill Response. 2010 11 24;1(1):1–2. 10.5365/wpsar.2010.1.1.007 23908872 PMC3729047

[3] Martin R, Fall IS. Field Epidemiology Training Programs to accelerate public health workforce development and global health security. Int J Infect Dis. 2021 9 7;S1201-9712(21)00655-X. (Article in press) 10.1016/j.ijid.2021.08.021 34518062

[4] Asia Pacific Strategy for Emerging Diseases and Public Health Emergencies (APSED III). Advancing implementation of the International Health Regulations (2005). Manila: World Health Organization Regional Office for the Western Pacific; 2017. Available from: https://iris.wpro.who.int/bitstream/handle/10665.1/13654/9789290618171-eng.pdf

[5] Responding to Typhoon Haiyan in the Philippines. Western Pac Surveill Response J. 2015; Suppl 1. Available from: https://ojs.wpro.who.int/ojs/index.php/wpsar/issue/view/26 10.5365/WPSAR.2015.6.4.HYN_026 PMC4710071 26767125

[6] Ushizawa H, Foxwell AR, Bice S, Matsui T, Ueki Y, Tosaka N, et al. Needs for disaster medicine: lessons from the field of the Great East Japan Earthquake. [Editorial]. West Pac Surveill Response. 2013 1 24;4(1):51–5. Available from https://ojs.wpro.who.int/ojs/index.php/wpsar/issue/view/5 10.5365/wpsar.2012.3.4.010 23908957 PMC3729104

[7] Western Pac Surveill Response J. COVID-19 collection. Manila: World Health Organization Regional Office for the Western Pacific; 2021. Available from: https://ojs.wpro.who.int/ojs/index.php/wpsar/issue/covid19collection

[8] Western Pac Surveill Response J. FETP Publications. Manila: World Health Organization Regional Office for the Western Pacific; 2021. Available from: https://ojs.wpro.who.int/ojs/index.ph/wpsar/issue/fetp_publications

[9] Pittman J, Stahre M, Tomedi L, Wurster J. Barriers and facilitators to scientific writing among applied epidemiologists. J Public Health Manag Pract. 2017 May-Jun;23(3):291–4. 10.1097/PHH.0000000000000433 27598712

[10] McPherson M, Counahan M, Hall JL. Responding to Typhoon Haiyan in the Philippines. West Pac Surveill Response. 2015 11 6;6 Suppl 1:1–4. 10.5365/wpsar.2015.6.4.HYN_026 26767125 PMC4710071

[11] Gocotano A, Counahan M, Belizario V, Hartigan-Go K, Balboa G, Go M, et al. Can you help me write my story? The institutional affiliations of authors of international journal articles on post-disaster health response. West Pac Surveill Response. 2015 11 6;6 Suppl 1:10–4. 10.5365/wpsar.2015.6.3.HYN_019 26767127 PMC4710074

